# Seasonal and geographical impact on the Irish raw milk microbiota correlates with chemical composition and climatic variables

**DOI:** 10.1128/msystems.01290-23

**Published:** 2024-03-06

**Authors:** Min Yap, Orla O'Sullivan, Paul W. O'Toole, Jeremiah J. Sheehan, Mark A. Fenelon, Paul D. Cotter

**Affiliations:** 1 Teagasc Food Research Centre, Cork, Ireland; 2 School of Microbiology, University College Cork, Cork, Ireland; 3 APC Microbiome Ireland, Cork, Ireland; 4 VistaMilk SFI Research Centre, Cork, Ireland; 5 Dairy Processing Technology Centre (DPTC), Limerick, Ireland; Colorado State University, USA

**Keywords:** milk microbiota, shotgun metagenomics, climate, milk composition

## Abstract

Season and location have previously been shown to be associated with differences in the microbiota of raw milk, especially in milk from pasture-based systems. Here, we further advance research in this area by examining differences in the raw milk microbiota from several locations across Ireland over 12 months, and by investigating microbiota associations with climatic variables and chemical composition. Shotgun metagenomic sequencing was used to investigate the microbiota of raw milk collected from nine locations (*n* = 241). Concurrent chemical analysis of the protein, fat, lactose, total solids, nonprotein nitrogen contents, and titratable acidity (TA) of the same raw milk were performed. Although the raw milk microbiota was highly diverse, a core microbiota was found, with *Pseudomonas_E*, *Lactococcus, Acinetobacter*, and *Leuconostoc* present in all samples. Microbiota diversity significantly differed by season and location, with differences in seasonality and geography corresponding to 11.8% and 10.5% of the variation in the microbiota. Functional and antibiotic resistance profiles also varied across season and location. The analysis of other metadata revealed additional interactions, such as an association between mean daily air and grass temperatures with the abundance of spoilage taxa like *Pseudomonas* species. Correlations were identified between pathogenic, mastitis-related species, fat content, and the number of sun hours, suggesting a seasonal effect. Ultimately, this study expands our understanding of the interconnected nature of the microbiota, environment/climate variables, and chemical composition of raw milk and provides evidence of a season- and location-specific microbiota.

**IMPORTANCE:**

The microbiota of raw milk is influenced by many factors that encourage or prevent the introduction and growth of both beneficial and undesirable microorganisms. The seasonal and geographical impacts on the microbial communities of raw milk have been previously seen, but the relationships with environmental factors and the chemical composition has yet to be investigated. In this year-long study, we found that while raw milk is highly diverse, a core microbiota was detected for Irish raw milk, with strong evidence of seasonal and geographical influence. We also found associations between groups of microorganisms, environmental factors, and milk composition, which expand current knowledge on the relationships between microbial and chemical composition and the climate. These results provide evidence for the development of a tool to allow for the prediction of raw milk quality and safety.

## INTRODUCTION

The composition of the microbiota of raw milk is of great importance as it can have a major effect on the quality of downstream dairy products. To produce high-quality dairy products, consistent, high-quality raw milk is required. This can be challenging as many factors influence the raw milk microbiota. Climate, environment, feed, season, lactation stage, storage conditions, and farm system, among others, have previously been identified as contributory factors (1–3). As studies on the microbiota of raw milk varied in the research questions asked and the parameters investigated, differing results have been observed. However, there is a consensus that a greater understanding of the patterns and influences of different factors on the raw milk microbiota can inform strategic interventions or practices to produce high-quality raw milk for further processing consistently.

High-throughput sequencing has been of considerable value when characterizing diverse milk microbiomes. It can use multiple omics methods coupled with other tools, such as modeling, to generate translatable results for implementation by the industry (4). While culture-based approaches tend to target specific bacterial taxa, using high-throughput sequencing techniques can provide a more comprehensive view of the entire microbial community in milk samples. Culture-based methods fail to capture the viable but nonculturable or non-readily culturable bacteria, an issue which can be overcome by using sequencing techniques (5). In addition to generating compositional data, shotgun metagenomic sequencing provides functional profiles, including information on the resistome (antibiotic resistance gene content) with additional quality control value.

Seasonal changes can impact the raw milk microbiota, with weather conditions and temperature particularly affecting the microbial composition of raw milk (6, 7). Such seasonal variations have also impacted the microbiota of downstream dairy products, such as cheese (8, 9). Several studies have reported differing levels of species richness and diversity between seasons and geographical locations (2, 10–13). Separately, changes in the chemical or nutritional composition of milk across seasons have also been reported, with various factors, including lactation stage and farm management systems having an impact (14). Protein and fat contents have reportedly displayed similar seasonal trends corresponding to stage of lactation in Ireland, Australia, and New Zealand, with fat and protein contents decreasing in spring to summer before increasing in autumn until the end of lactation in winter (15). However, no attempts have been made to correlate chemical composition with climatic factors or the raw milk microbiota. Therefore, this study aimed to investigate the fluctuations in the microbiota of raw milk collected from farms operating a pasture-based system across different regions of Ireland over 12 months, and to test for interactions between the milk microbiota and climatic data and milk chemical composition data.

## RESULTS

### The Irish raw milk microbiota is highly diverse but has a core microbiota

Two hundred forty-one raw milk samples collected from nine different locations across Ireland were sampled over a year. An average of 3,890,014 (± 2,258,584) high-quality paired-end reads corresponding to 0.52 Gbp were generated per sample. An average of 68.6% of metagenomic reads were bovine genome reads (range 0.4% to 98.7%). This resulted in an average of 1,121,982 (± 875,900) reads that were assigned as microbial reads (mean 31.4%, range 1.35% to 99.61%) and used for taxonomic and functional characterization. A total of 1,112 genera and 3,332 species were detected in samples, with 26 genera and 49 species detected in samples at an average of at least 1% relative abundance and, thus, a high proportion of genera and species were detected at levels lower than 1%. Species identified were classified into five categories (environment, host, pathogen, spoilage, technologically relevant), based on previous literature (Table S1). Taxa were assigned to the category that was indicated by most of the literature, despite some taxa potentially able to be classified into more than one category. A total of 694 metagenome-assembled genomes (MAGs) were assembled, of which 153 were high-quality MAGs that were classified into 23 different genera and 35 different species, and with 7 MAGs that could only be classified to genus level (Table S2). Among the high-quality MAGs recovered, we identified 19 unique taxa that were not detected based on the classification of short reads (Table S2).

**Fig 1:**
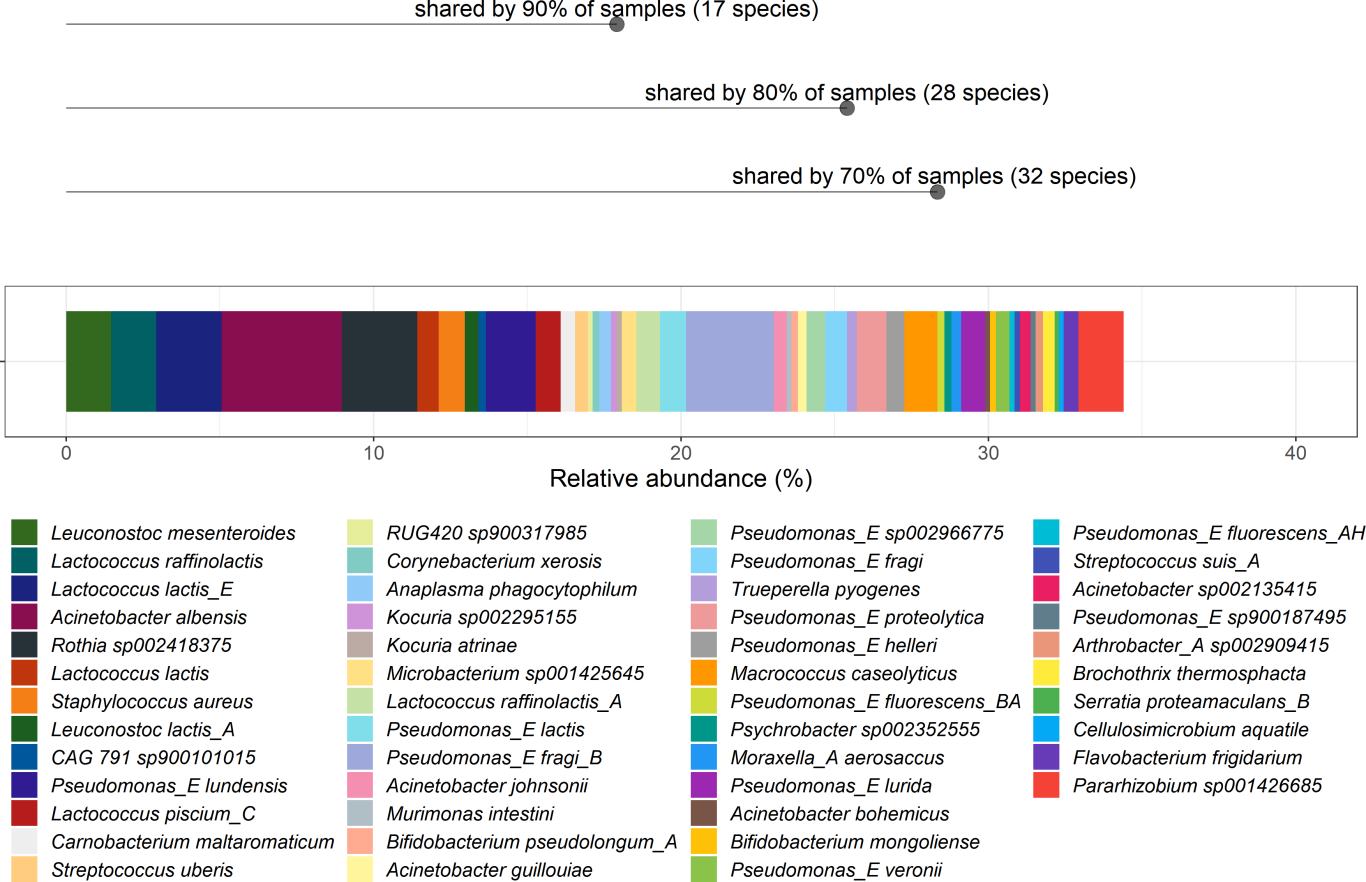
Species-level composition of the bulk milk microbiota, comprising species with an average relative abundance of greater than 1%, that account for 34.4% of total average relative abundance.

Despite the considerable inter-sample variation, a core microbiota was detectable, where taxa were present in all samples. In this study, the genera *Pseudomonas_E*, *Lactococcus, Acinetobacter,* and *Leuconostoc* present in all samples, contributing to a total of 34.2% (17% *Pseudomonas_E*, 7.5% *Lactococcus*, 7.2% *Acinetobacter*, 2.5% *Leuconostoc*) of the relative abundance of the bulk milk microbiota. The genus *Lactococcus* had the greatest number of species (*n* = 4) present in at least 90% of the samples, and these four species (*Lactococcus lactis, L. lactis_E, Lactococcus piscium_C* and *Lactococcus raffinolactis*) together accounted for an average of 5% of the milk microbiota (Fig. 1). As well as being the most abundant at genus level, there were also more species of *Pseudomonas* detected than for any other genus, i.e., 12 species of *Pseudomonas* were detected in 73% of the samples (178/244 samples), with *Pseudomonas_E lundensis*, *Pseudomonas lactis,* and *Pseudomonas fragi_B* being the three most commonly detected species from the genus *Pseudomonas*. Four species of *Acinetobacter* were identified, with *Acinetobacter albensis* present in 97% of the samples (237/244), accounting for 3.9% relative abundance. Two species of *Leuconostoc* were present, *Leuconostoc lactis_A* and *Leuconostoc mesenteroides*, in 95% and 98% of the samples, respectively, accounting for 1.9% of relative abundance. It was also noted that *Bifidobacterium pseudolongum_A* and *Staphylococcus aureus* were present in 83% (202/244 samples) and 96% of all samples (234/244 samples) and contributed an average relative abundance of 0.21% and 0.86%, respectively. *Pararhizobium* sp001426685 was only present in 17 samples (7% of all samples) but was one of the top 10 most abundant species detected, accounting for an average of 1.46% relative abundance (Fig. 1). Similar patterns were evident when MAGs were analyzed, in that *Pseudomonas_E*, *Lactococcus, Acinetobacter,* and *Leuconostoc* had the greatest numbers of high-quality MAGs recovered (Table S2). Interestingly, four additional species of *Lactococcus* were identified compared to the short read classification reported above. Twelve MAGs of *Lactococcus cremoris*, nine MAGs of *Lactococcus laudensis*, two MAGs of *Lactococcus carnosus,* and one MAG of *Lactococcus petauri* were recovered in addition to the species mentioned above (*L. lactis* and *L. raffinolactis*). For the MAGs classified as *Pseudomonas*, three additional species were recovered (*Pseudomonas bubulae*, *Pseudomonas paracarnis,* and *Pseudomonas saxonica*). Conversely, more species were classified as *Acinetobacter* based on the short read data compared to the recovered *Acinetobacter* MAGs, in that only *A. albensis* (25 MAGs) and *Acinetobacter* sp002135415 (1 MAG) were recovered. Lastly, MAGs corresponding to *Leuconostoc mesenteroides* (16 MAGs) and *Leuconostoc rapi* (1 MAG) were also recovered.

### Both season and location influence the raw milk microbiota

**Fig 2:**
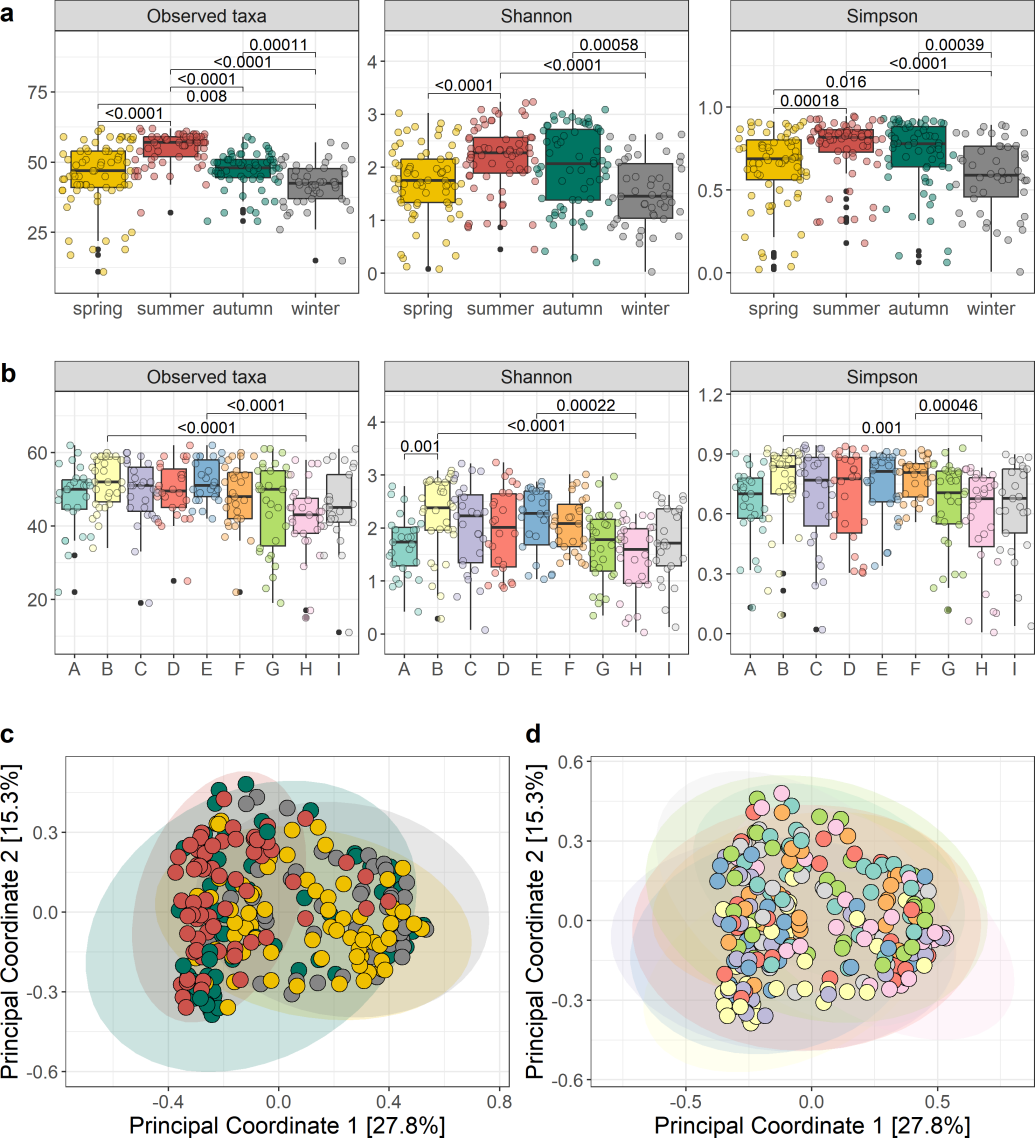
Diversity of the raw milk microbiota, with the observed genera, Shannon and Simpson alpha diversity metrics by (**a**) season and (b) sampling location across the 12-month sampling period. Bray-Curtis principal coordinate analysis (PCoA plots), by (c) season and (d) sampling location with ellipsis representing clustering by season and sampling location, respectively.

The alpha diversity (observed taxa, Shannon and Simpson metrics) of the raw milk microbiota differed significantly across seasons, with significantly greater numbers of taxa observed in the summer months and significantly fewer taxa detected in the winter months (*P* < 0.05) (Fig. 2a). According to the Shannon and Simpson metrics, the diversity during the summer and autumn months was significantly higher than for spring and winter (*P* < 0.05). Across the nine sampling locations, there were fewer taxa observed at locations H and I, while Shannon and Simpson diversity indices were generally lower across locations A, G, and H compared to the other locations (Fig. 2b). The beta diversity analysis of the raw milk microbiota revealed significant separation of samples across seasons, with each season distinct from the others [permutational multivariate analysis of variance (PERMANOVA), *P* < 0.05] (Fig. 2c; Table 1). The beta diversity difference between sampling locations was also statistically significant (PERMANOVA, *P* < 0.05) (Table 1), with sampling locations A and H being the most distinct (Fig. 2d). Both season and location interacted significantly with the variation in microbiota composition (*P* < 0.05), and accounted for 11.8% and 10.5% of the variation, respectively.

**TABLE 1:** Sample metadata and the degree to which their variation explains differences in the taxonomic composition and functional profiles of the raw milk microbiota, calculated using PERMANOVA (adonis, vegan)

Model or variable	Variation explained by (*R^2^*)	*P^a^*
Taxonomic composition		
Location	10.523	0.001 ***
Season	11.802	0.001 ***
Mean temperature	1.089	0.001 ***
Total rainfall	0.812	0.01 **
Grass minimum temperature	1.068	0.006 **
Mean wind speed	0.336	0.328
Sun hours	0.635	0.042 *
Functional profile		
Location	0.0976	0.001 ***
Season	0.124	0.001 ***
Mean temperature	0.00867	0.052
Total rainfall	0.00695	0.106
Grass minimum temperature	0.00431	0.256
Mean wind speed	0.00231	0.506
Sun hours	0.0102	0.04 *

^*a*^ *** *P* ≤ 0.001, ** *P* ≤ 0.01, * *P* ≤ 0.05.

**Fig 3:**
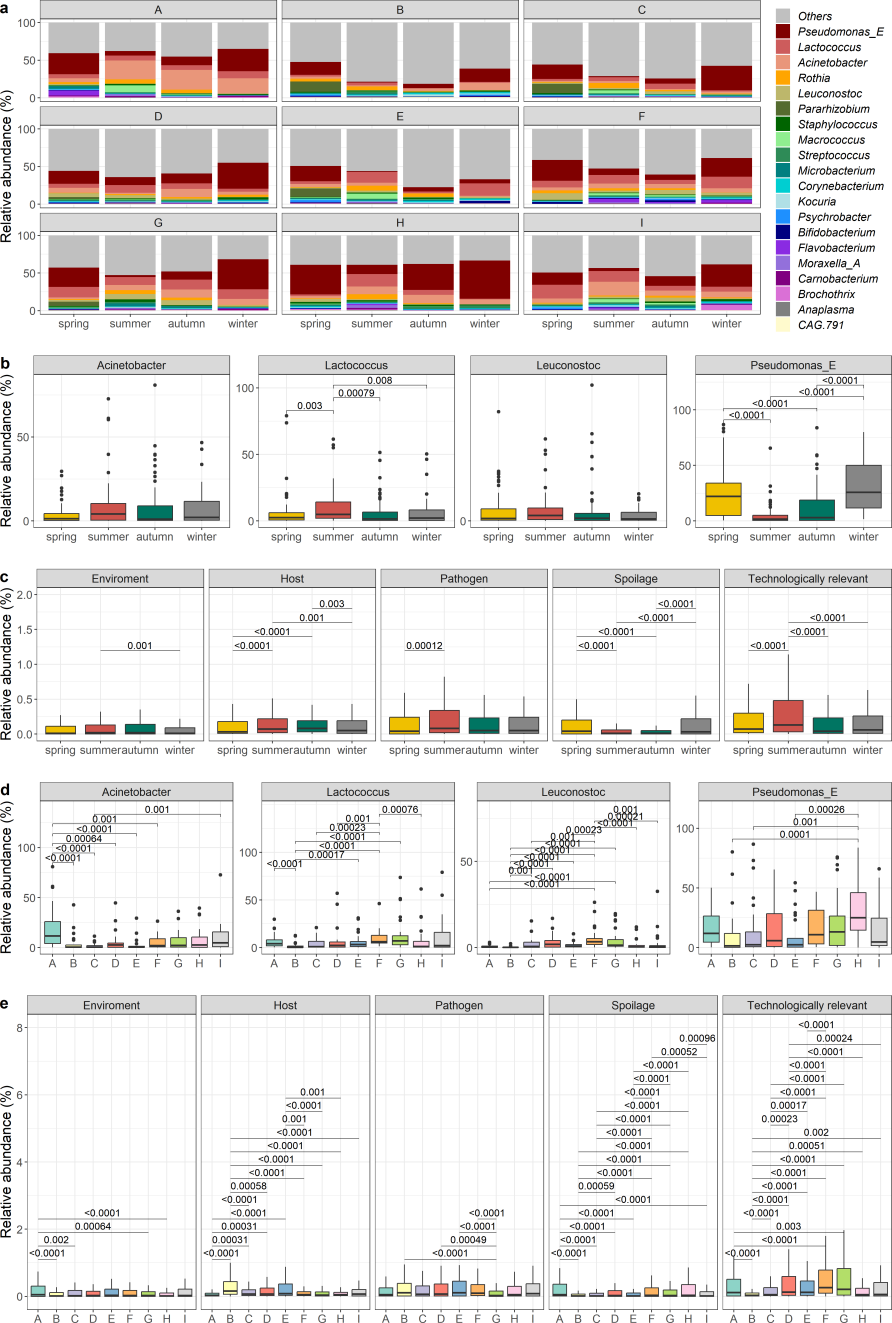
(a) Genus-level taxonomic composition of the top 20 most abundant genera of the raw milk microbiota across seasons by sampling location, and the changes in the core genera (b) and in species grouped into five categories (c) by season, and the changes in the core genera (d) and in species grouped into five categories (e). The five categories are environment-related, host-related, pathogenic, spoilage, and technologically relevant species and the species in each category are in Table S1.

Significant differences in the relative abundances of 16 of the 20 most abundant genera were found between seasons (Fig. 3a; Table S3; Fig. S1). Specifically, with respect to the core genera, the relative abundance of *Acinetobacter* was higher in summer compared to spring, though not statistically significant, *Lactococcus* was significantly more abundant in summer than the other seasons, *Pseudomonas_E* was significantly more abundant in winter and spring compared to summer and autumn (*P* < 0.05), while no seasonal differences in the relative abundances of *Leuconostoc* were observed (Fig. 3b). For other taxa, samples collected in the summer contained significantly higher relative abundances of *Anaplasma*, *Flavobacterium*, *Macrococcus*, *Moraxella_A*, *Rothia*, *Staphylococcus,* and *Streptococcus*, and significantly lower relative abundances of CAG 791, a genus of the family *Lachnospiraceae*, compared to the other three seasons (*P* < 0.05) (Fig. 3a). Spring samples had significantly higher relative abundances of *Pararhizobium* and *Psychrobacter* compared to the other seasons, while relative abundances of *Microbacterium, Rothia*, and *Brochothrix* were significantly lower in winter and autumn samples, respectively. Both winter and spring samples had significantly higher relative abundances of *Bifidobacterium* compared to autumn and summer, but significantly lower relative abundances of *Macrococcus* and *Moraxella_A* compared to autumn samples. *Carnobacterium* was detected in significantly higher relative abundances in summer and winter samples than those collected in spring and autumn.

Species identified were assigned into five categories (environment, host, pathogen, spoilage, technologically relevant), based on previous literature (Table S1). Those related to the environment (soil, hay, silage, bedding) had significantly lower relative abundances in winter compared to summer, while species related to spoilage were significantly higher in spring and winter compared to summer and autumn (*P* < 0.05) (Fig. 3c), which mostly consisted of *Pseudomonas* species (Table S1). Summer milk samples had significantly higher relative abundances of environment-related, host-related, pathogenic, and technologically relevant species (*P* < 0.05) (Table S1). Additional analysis was performed to determine if any particular species was significantly associated with a particular season and, while no particular species was specifically associated with autumn samples, those collected in spring and summer were each significantly associated with six unique species and those collected in winter were associated with seven species (*P* < 0.05). Spring was associated with the presence of *Pararhizobium* sp001426685, *Pseudomonas_E veronii*, *Psychrobacter* sp002352555, *Bifidobacterium pseudolongum_A*, *Pseudomonas_E* sp900187495, and *Cellulosimicrobium aquatile*; summer samples were associated with *Anaplasma phagocytophilum, Lactococcus lactis*, *Macrococcus caseolyticus*, *Moraxella_A aerosaccus*, *Murimonas intestini,* and *Rothia* sp002418375; and winter samples were associated with CAG 791 sp900101015, *Kocuria atrinae*, *Pseudomonas_E fragi*, *Pseudomonas_E fragi_B*, *Pseudomonas_E lactis*, *Pseudomonas_E lurida,* and *RUG420* sp900317985.

Significant differences in the relative abundances of 16 of the top 20 genera were also identified across sampling locations (Fig. 3a; Table S4). Among the core genera, the relative abundances differed across sampling locations, with relative abundances of *Acinetobacter* being highest in samples from location A (19.34%), followed by samples from location I (10.06%) (Fig. 3d). *Lactococcus* abundances were highest in samples from G (12.77%), followed by those from I (10.97%) and F (10.19%). Location F had the highest relative abundance of *Leuconostoc* (5.22%), while H had the highest for *Pseudomonas_E* (32.59%) (Fig. 3d). In particular, samples from location A had significantly higher relative abundances of *Acinetobacter*, *Brochothrix,* and *Carnobacterium* and significantly lower relative abundances of *Bifidobacterium* compared to the other locations (*P* < 0.05). Location B samples had a significantly lower abundance of *Lactococcus* (1.85%) compared to all other locations except for location C (4.2%) (*P* < 0.05), and significantly higher relative abundances of *Kocuria* compared to samples from A, F, and G (*P* < 0.05). Location F had a significantly higher relative abundance of *Leuconostoc* except for samples from locations D and G (*P* < 0.05). Relative abundances of *Pararhizobium* were lowest in location H (0.001%) compared to other locations, with B (4.54%), C (3.13%), and E (4.38%) having significantly higher relative abundances (*P* < 0.05). *Psuedomonas_E* was higher in H (32.59%) compared to all locations, and was significantly higher than B (10.85%), C (14.29%), E (10.29%), and I (15.02%) (*P* < 0.05). When the species identified were classified into categories, significantly higher relative abundances of environment-related species were detected in milk from A (*P* < 0.05). In comparison, B and E had significantly higher relative abundances of host-related species (*P* < 0.05) (Fig. 3e; Table S1). Relative abundances of pathogens were significantly lower in milk from sampling location G than in other locations (*P* < 0.05). For species related to spoilage, significantly higher relative abundances were found in milk from locations A and H, and significantly lower relative abundances in milk from B (*P* < 0.05). For technologically relevant species (mainly from the genera *Bifidobacterium*, *Lactococcus,* and *Leuconostoc*), significantly higher relative abundances were found in milk from F and G with significantly lower relative abundances found in milk from B (*P* < 0.05) (Fig. 3e). Further analysis found significant associations between six species and four sampling locations (*P* < 0.05). *Acinetobacter albensis* was significantly associated with A, while CAG 791 sp900101015 and *Kocuria atrinae* were associated with B, *Leuconostoc lactis_A* was associated with D and *Leuconostoc mesenteroides*, *Lactococcus raffinolactis_A,* and *Bifidobacterium mongoliense* were associated with F. No particular species were correlated with sampling locations C, E, G, H, and I.

### Functional and antibiotic resistance profiles differed by season and sampling location

**Fig 4:**
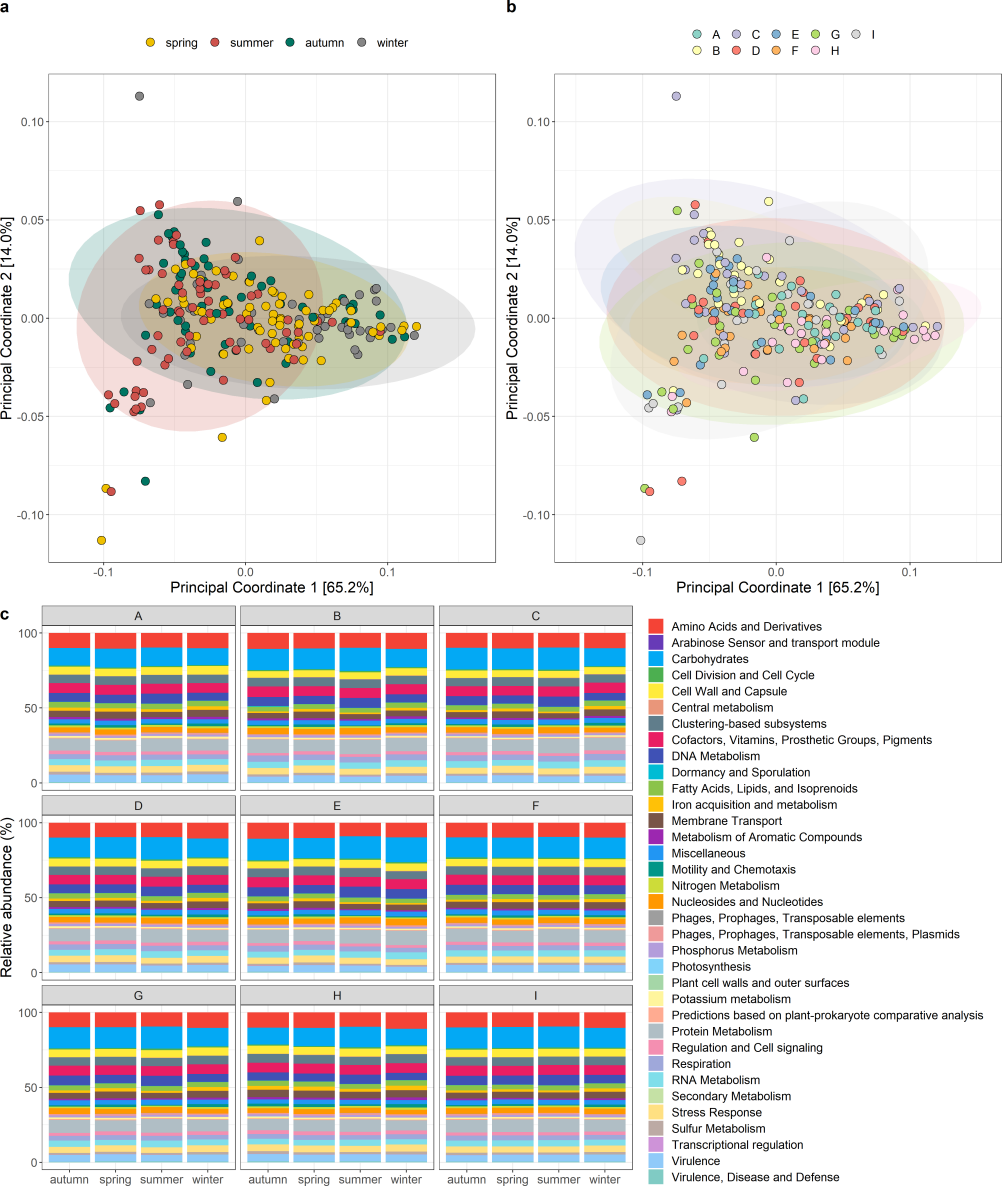
Functional characteristics of the raw milk microbiota based on SUPER-FOCUS analysis. Beta diversity Bray-Curtis principal coordinate analysis plots by (a) season and (b) location, and (c) the subsystem level 1 functions by season across the sampling locations.

Season and location also impacted the predicted functional potential of the raw milk microbiota, with differences found in subsystem level 1 functions. Similar to taxonomic characteristics, the beta diversity of functional profiles was significantly different across seasons, with each season being distinct from the others, with the exception of spring and winter (PERMANOVA, *P* < 0.05) (Fig. 4a; Table 1). With respect to sampling locations, significant differences were observed between locations (PERMANOVA, *P* < 0.05) (Table 1), with A and H differing from B, C, E, and F, and H and I clustering separately from I (Fig. 4b). Differences were also observed between abundances of subsystem level 1 functions, with 14 and 19 functions significantly different by season and location, respectively (Table S3 and S4; Fig. 4c).

**Fig 5:**
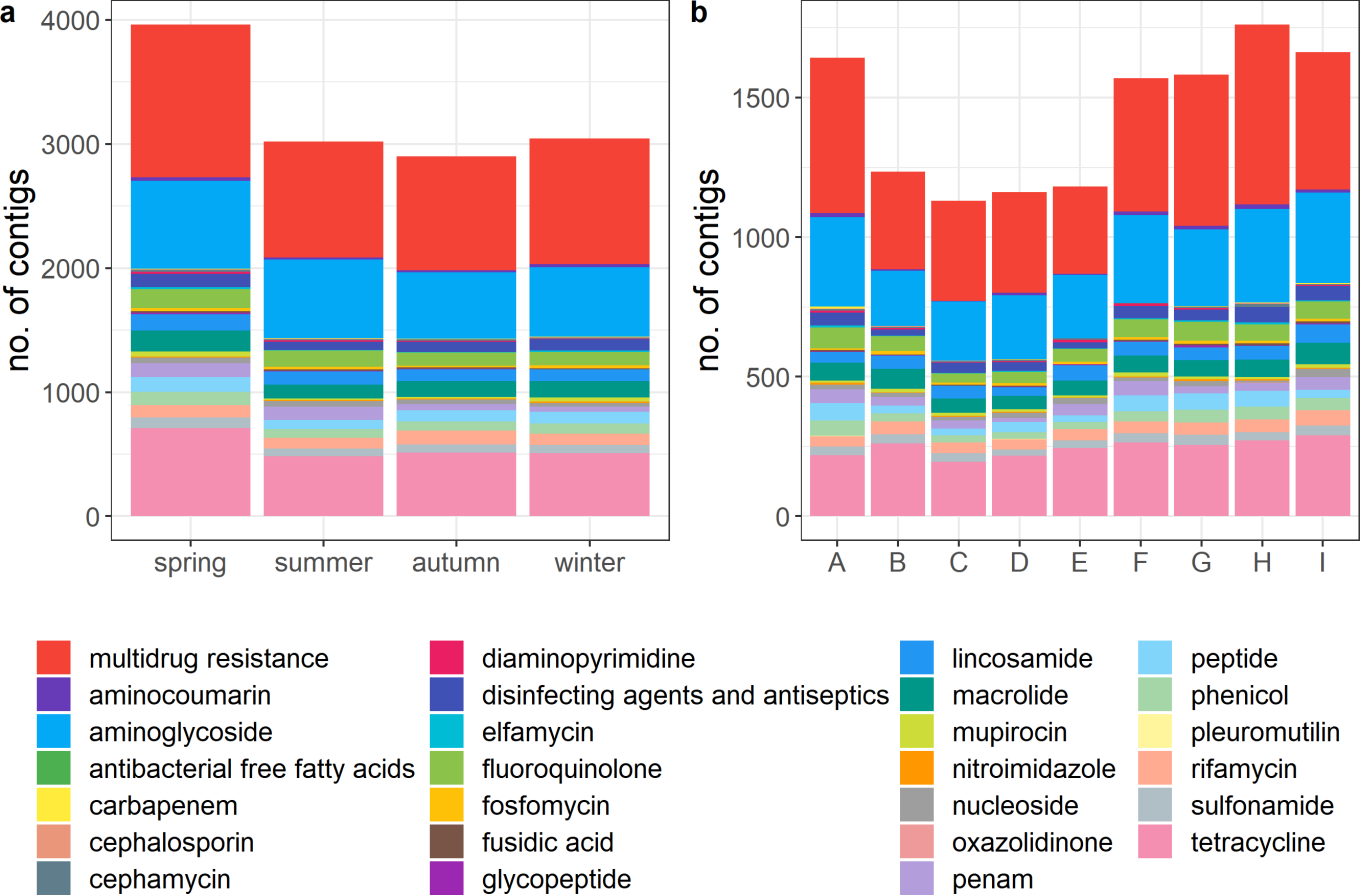
Composition of the number of contigs mapped to genes from different drug resistance classes of the raw milk microbiota, by (a) season and (b) sampling location.

The resistome of the raw milk microbiota was predicted using Resistance Gene Identifier (RGI) and the number of contigs mapped to antibiotic resistance genes classified were categorized into different drug classes (Fig. 5). Genes from a total of 27 drug class resistance mechanisms were detected. The greatest proportion of genes belonged to the multidrug resistance category (31.7%), followed by genes encoding resistance to aminoglycosides (18.8%) and tetracycline (17.1%). Seasonal variation was evident, with a significantly greater number of contigs of relevance in samples taken in spring compared to the other seasons (*P* < 0.05). Although not statistically significant, differences between sampling locations were seen, with location I having the highest number of mapped contigs (1,761 contigs) and C having the lowest (1,130 contigs).

### The raw milk microbiota correlated with climatic factors and chemical composition

**Fig 6:**
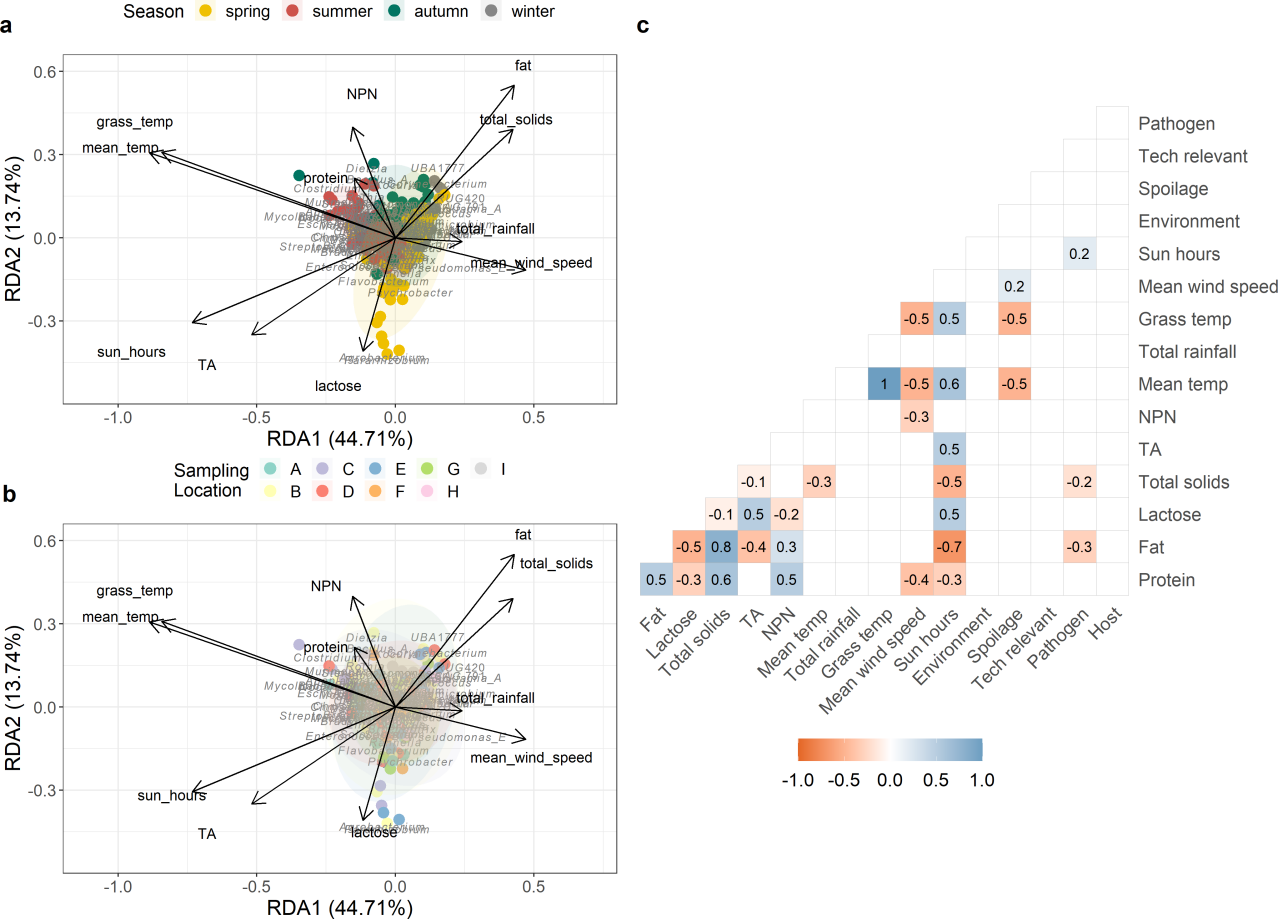
Correlation of climatic factors and chemical data and the raw milk microbiota. RDA of the relationship between the climatic factors and chemical data and the relative abundance of the top 20 genera of the raw milk microbiota, as seen by (a) season and (b) location, and (c) Pearson correlation matrix of raw milk chemical composition, climatic factors with the species detected in the raw milk microbiota grouped into the five different categories (Table S1).

Data from the analysis of the chemical composition of raw milk and climatic data across the sampling period are summarized in Tables 2 and 3. Redundancy analysis (RDA) was performed to investigate correlations between several climatic factors (mean temperature, total rainfall, grass temperature, mean wind speed, and number of hours of sunshine) and the raw milk microbiota as well as the relationship between the microbiota and chemical composition [protein content, fat content, lactose content, total solids content, titratable acidity (TA), nonprotein nitrogen (NPN) content] (Fig. 6a and b). The Pearson’s correlation coefficient was calculated, and statistical significance was tested (Fig. 6c). Redundancy analysis showed that the climatic variables explained the variation in the bulk milk microbiota, with a constrained proportion of 14.37%, and with all variables statistically significant (*P* < 0.05). When visualized by season, logical patterns were seen, with mean wind speed and total rainfall related to spring and winter and mean temperature and grass temperature correlated to summer (Fig. 6a). Of note, *Pararhizobium* and *Agrobacterium* abundances were found to be related to spring and sampling locations B, C, and E, but these genera were also found to be associated with the lactose content of milk (Fig. 6a and b). *Pseudomonas_E* abundance was related to winter and spring seasons. It was significantly associated with the increase of the mean wind speed and total rainfall and the decrease of mean and grass temperature (Fig. 6a), which was confirmed with a significant correlation found with these same variables, with *Pseudomonas* spp. under spoilage-related taxa (Fig. 6c; Table S1). No other visible strong relationships in any particular sampling location were detected (Fig. 6b). From Pearson’s correlation coefficient analysis, several associations were apparent between the climatic variables, chemical composition, and the raw milk microbiota. Raw milk’s components (protein, fat, lactose, total solids, TA, NPN contents) were highly correlated as shown in Fig. 6c, where significant positive and negative correlations were seen between the variables. No significant correlations were found between environment, host-related, and technologically relevant taxa, and chemical composition and climatic variables (Fig. 6c). Spoilage-related species were negatively correlated with mean and grass minimum temperatures and positively correlated with mean wind speed (Fig. 6c), suggesting low-temperature spoilage in raw milk. Pathogenic species related to mastitis or disease were significantly correlated with measures of fat, total solids content, and number of sun hours (Fig. 6c).

**TABLE 2:** Sample size, chemical composition, and climatic variables of Irish raw milk across seasons during the sampling period

	Season			
	Spring	Summer	Autumn	Winter
*N*	69	66	63	46
Chemical composition				
Protein (%)	3.349 ± 0.1	3.455 ± 0.1	3.832 ± 0.2	3.391 ± 0.2
Fat (%)	4.15 ± 0.2	3.997 ± 0.3	4.578 ± 0.3	4.27 ± 0.3
Lactose (%)	4.82 ± 0.1	4.69 ± 0.1	4.52 ± 0.2	4.605 ± 0.2
Total solids (%)	13.04 ± 0.4	12.758 ± 0.4	13.565 ± 0.6	13.105 ± 0.4
TA (%)	0.16 ± 0.01	0.16 ± 0.01	0.15 ± 0.01	0.15 ± 0.01
NPN (%)	0.026 ± 0.002	0.027 ± 0.003	0.031 ± 0.004	0.026 ± 0.004
Climatic variables				
Mean temperature (°C)	6.7 ± 1.3	15.6 ± 1.2	11.8 ± 2.7	7.4 ± 0.7
Total rainfall (mm)	83.1 ± 34.1	58.3 ± 16.8	102.3 ± 41.1	96.9 ± 32.7
Grass minimum temperature (°C)	−7.3 ± 1.4	4.2 ± 1.5	0.1 ± 2.4	−4.9 ± 1.0
Mean wind speed (knot)	6.2 ± 0.4	5.0 ± 0.4	5 ± 0.4	7.6 ± 1.8
Hours of sunshine (h)	11.1 ± 2.0	15.0 ± 1.0	9.7 ± 1.0	7.8 ± 0.9

**TABLE 3:** Sample size, chemical composition, and climatic variables of Irish raw milk across locations during the sampling period

	Location								
	A	B	C	D	E	F	G	H	I
*N*	27	30	27	26	25	27	30	27	25
Chemical composition									
Protein (%)	3.50 ± 0.2	3.46 ± 0.2	3.51 ± 0.2	3.52 ± 0.3	3.45 ± 0.3	3.34 ± 0.2	3.50 ± 0.2	3.34 ± 0.1	3.54 ± 0.3
Fat (%)	4.23 ± 0.6	4.22 ± 0.3	4.32 ± 0.3	4.24 ± 0.4	4.24 ± 0.4	4.09 ± 0.2	4.28 ± 0.4	4.15 ± 0.2	4.27 ± 0.4
Lactose (%)	4.65 ± 0.2	4.62 ± 0.2	4.64 ± 0.3	4.69 ± 0.1	4.75 ± 0.2	4.58 ± 0.2	4.67 ± 0.2	4.81 ± 0.1	4.67 ± 0.2
Total solids (%)	13.05 ± 0.6	13.09 ± 0.4	13.1 ± 0.5	13.09 ± 0.5	13.12 ± 0.5	12.72 ± 0.4	13.12 ± 0.5	13.03 ± 0.3	13.3 ± 0.8
TA (%)	0.16 ± 0.012	0.15 ± 0.012	0.15 ± 0.013	0.16 ± 0.012	0.15 ± 0.014	0.15 ± 0.01	0.15 ± 0.015	0.15 ± 0.008	0.15 ± 0.01
NPN (%)	0.028 ± 0.004	0.027 ± 0.004	0.027 ± 0.004	0.028 ± 0.004	0.027 ± 0.004	0.027 ± 0.004	0.027 ± 0.004	0.027 ± 0.002	0.028 ± 0.007
Climatic variables									
Mean temperature (°C)	9.8 ± 3.9	8.4 ± 3.8	8.4 ± 3.9	9.1 ± 3.8	9.8 ± 3.8	9.8 ± 3.9	8.4 ± 3.8	9.8 ± 3.9	8.4 ± 3.9
Total rainfall (mm)	83.1 ± 36.7	62.9 ± 36.6	62.9 ± 37.3	73 ± 36.4	62.9 ± 36.5	83.1 ± 35.8	62.9 ± 36.9	83.1 ± 36.9	83.1 ± 35.3
Grass minimum temperature (°C)	−3.9 ± 4.3	−4.2 ± 4.3	−3.9 ± 4.3	−3.9 ± 4.3	−3.9 ± 4.4	−3.9 ± 4.4	−4.2 ± 4.2	−3.9 ± 4.3	−3.9 ± 4.4
Mean wind speed (knot)	5.2 ± 1.2	5.2 ± 1.1	5.2 ± 1.1	5.4 ± 1.2	5.2 ± 0.9	5.2 ± 1.2	5.2 ± 1.2	5.6 ± 1.2	5.2 ± 1.3
Hours of sunshine (h)	11.1 ± 2.9	11.1 ± 3.0	11.1 ± 3.1	11.1 ± 2.8	11.1 ± 2.7	11.1 ± 3.0	10.7 ± 3.1	11.1 ± 3.1	10.2 ± 2.9

## DISCUSSION

In this study, raw milk was found to have a diverse microbiota, which differed according to season and location, as reported previously by us and by others (10, 12, 13). A large proportion of taxa were present at less than 1% relative abundance at both species and genus level, consistent with previous studies (2, 16). Despite this, a core microbiota was observed, with *Acinetobacter*, *Lactococcus*, *Leuconostoc,* and *Pseudomonas* identified in all raw milk samples. *Pseudomonas*, *Lactococcus,* and *Acinetobacter* have been commonly detected in raw milk samples using high-throughput sequencing (6, 11). While less abundant, *Leuconostoc* has been found to be prevalent in Irish bovine milk and is a potentially beneficial microbe, with some species contributing to the organoleptic properties of resultant dairy products (16). Seasonality and geography, in particular, have been found to correlate with this diversity.

The bacterial communities and the associated functional profiles and resistome of samples differed across each season. Samples from summer were observed to have the most diverse community with the highest number of taxa observed. Significant differences in the bacterial community structure between sampling seasons have also been found by Kable et al. (2) and Guo et al. (10), with similar trends of greater evenness and richness in the spring and summer months compared to autumn or winter months. This pattern is, however, different from our previous study, which noted a higher alpha diversity in samples taken in November (12). These differences could be explained by a variety of factors such as weather, animal health, feed, or farm environment (17). In summer, abundances of environment host-related, pathogenic, and technologically relevant species were higher. This has also been reported in several studies where *Acinetobacter* (environment-related), *Lactococcus* and *Bifidobacterium* (frequently technologically relevant), and *Corynebacterium* and *Streptococcus* (potentially skin/pathogenic/mastitis-related) species were more abundant in warmer months (2, 6, 13). Environment-related taxa were detected in significantly lower abundances in winter, reflecting Irish farming practices, where cows are kept indoors in winter, which has been previously reported to impact the raw milk microbiota (18). Host-related species like *Anaplasma phagocytophilum* and *Murimonas intestini* have been previously detected in bulk tank milk (12). In spring and winter, spoilage-related taxa like *Pseudomonas* spp., *Brochothrix thermosphacta,* and *Flavobacterium frigidarium* were in greater abundance, which is expected due to their psychrotrophic nature (3, 17). The longer storage times at farm level due to the decreased frequency of milk collection in winter could potentially account for the increased abundance of *Pseudomonas* in raw milk in winter. Overall, the fluctuating abundances of taxa across the year show the influence of seasonality on the raw milk microbiota. The influence of seasonality on the chemical composition of raw milk was similar to that previously reported, with total solids higher in autumn than summer, fat content higher in autumn, protein content higher in autumn and winter, and slight seasonal variations found for lactose based on lactation period (14, 19). In terms of the resistome, spring samples were found to have a significantly higher number of contigs harboring antibiotic resistance genes. While studies have been done on the raw milk resistome, the influence of seasonality has not been investigated. As the resistome of any environment is linked to microbiota composition, more analysis is needed to link species with antibiotic resistance genes present to understand the implications of these results (20).

This study also found significant differences in diversity and taxonomic composition across sampling locations, with certain taxa associated with specific locations. This suggests the presence of location-specific microbial communities in raw milk, where environmental factors at each sampling location influence the raw milk microbiota. Environmental and farming practices have been suggested as possible reasons for the differences in abundances across locations (21, 22). Variations in host- and environment-related species, in addition to the association of certain species with four out of the nine locations, support the hypothetical existence of a location-specific microbiota, but more data over a longer period of time are needed to establish this definitively. Regarding the resistome, in line with previous reports, the number of contigs harboring antibiotic resistance genes differed across sampling locations (23).

When comparing the influence of both season and location, similar findings to those reported by Guo et al. (10) were noted in that seasonal variations were associated with more significant differences in the raw milk microbiota than differences in geographical location. More specifically, although both factors were associated with statistically significant differences, seasonality explained a greater proportion of variation compared to location for both the taxonomic composition and the functional profiles. Many other variables contribute to differences in the milk microbiota that were not examined in this study but have been reported by others. These include animal housing regime, milking technology employed, animal health, diet, storage, and transport, among others (2, 3, 17, 18, 24–26). In this study, climatic variables were found to significantly explain the variation in the raw milk microbiota through PERMANOVA and redundancy analysis, a pattern which has been previously reported (6). Indeed, all variables except mean wind speed were found to significantly influence variation in the milk microbiota. This is consistent with findings where climatic variables were the most frequent factors impacting production level and comfort of grazing animals (27). Mean daily and grass temperature seemed to have had a greater impact, correlating with taxa-related spoilage like *Pseudomonas* species, implying the possibility of the influence of the time spent indoors by herds on the raw milk microbiota. Shathele (28) previously found that temperature was related to mastitis occurrence, with different bacteria causing mastitis in warm and cold months. Temperature or heat stress has also been found to affect cows, which in turn affect the composition of milk and the occurrence of mastitis (29). Relationships between fat content and hours of sun and the presence of pathogenic species were seen in our study. This suggests possible seasonal effects on both the fat content and microbiota of raw milk. Albonico et al. (30) found that the fat composition of milk was strongly associated with milk microbiota richness. Therefore, from the current study, climatic variables were seen to interact with and apparently influence the chemical and microbiological characteristics of raw milk, similar to results reported by Molina Benavides et al. (27), which could be useful in predicting raw milk quality and safety, though further work has to be done. Understanding the interactions between climate, chemical, and microbial composition would be largely beneficial in decision making for industry, regulatory bodies, and consumers of raw milk. Moreover, these results from such community-based sequencing studies can be the starting point for an omics-based model to predict raw milk quality and safety, which would enable the industry to make more timely quality and safety decisions.

This study is one of the few studies that employed shotgun metagenomic sequencing on the raw milk microbiota. Many previous studies have used amplicon sequencing or culture-based methods to understand the composition of milk across farms and seasons (2, 6, 7, 13). However, the current study had several limitations, one of which is the reliance on sequencing data alone, which meant that only relative abundances (rather than absolute abundances) of taxa could be inferred, which adds some bias as a rise in one taxon could mean the decrease in another. Furthermore, all microbial DNA present in the sample were sequenced, which included both viable and potentially some nonviable microorganisms. In this analysis, the focus was on bacterial communities present in raw milk as these are known to have the biggest impact on milk quality and safety; therefore, we do not report on prokaryotes. Moreover, sampling of raw milk from the same farms across multiple years would help to validate findings on the core microbiota and season- and location-specific nature of the raw milk microbiota. The impact of the raw milk microbiota across seasons and locations on the final product would be an interesting area of investigation for future studies.

This study clearly adds to the current research on the effect of seasonality and geography on the microbiota and resistome of raw milk. Additionally, the effects of climatic variables on the chemical properties and composition of the raw milk microbiota show how interconnected all these factors are, which has potential for use in predicting raw milk quality and safety through modeling. Despite the detection of a highly diverse milk microbiota, a core microbiota exists and potentially season- and location-specific taxa, but further sampling needs to be done to determine if these patterns hold true over an even longer period.

## MATERIALS AND METHODS

### Sample collection and preparation

Raw bovine milk samples (200 mL) were collected from silos from nine locations across Ireland weekly from March 2021 to March 2022. The samples were collected over 2 days, transported under refrigeration and stored at 4°C, to mimic conditions of their storage in bulk tanks or silos, for a maximum of 48 h before processing of all samples together. Samples were prepared as follows: 30 mL of the bovine milk sample was centrifuged at 4,500 × *g* for 20 min at 4°C. After centrifugation, the cream and supernatant were discarded, and the pellets were subjected to two washing steps, whereby the pellets were resuspended in sterile phosphate-buffered saline (PBS) and centrifuged at 13,000 × *g* for 1 min, after which the supernatant was discarded, and the pellet was stored at −20°C before DNA extraction. The chemical composition of the 100 mL of milk samples was determined by the Dairy Processing Technology Centre analytical staff at the Technical Services lab at the Teagasc Food Research Centre. Kjeldahl analysis was used to determine protein and NPN contents. Rose Gottlieb method was used to determine fat content, and the CEM SMART Trac II (CEM, Matthews, NC, USA) was used to measure the total solids content. Polarimetry was used to determine the lactose content, and titration was used to determine TA in raw milk samples. Monthly climate data for the sampling locations relating to mean temperature (°C), total rainfall (mm), grass minimum temperature (°C), mean wind speed (knots), and sunshine (hours of sun) were retrieved from the Irish Meteorological Service website (www.met.ie) (Table S2). The months of March, April, and May were classified as spring, June, July, and August as summer, September, October, and November as autumn and December, January, and February as winter.

### DNA extraction

Samples were subjected to DNA extraction using the MolYsis complete5 kit (Molzym GmBH & Co. KG, Bremen, Germany), with 50 µL of DNA eluted for downstream sequencing. The MolYsis kit was used to improve microbiota characterization by significantly enhancing the microbial sequencing depth of milk samples (31). gDNA was quantified using the Qubit dsDNA HS assay kit (Invitrogen) and stored at −20°C before library preparation.

### Illumina DNA library preparation and shotgun metagenomic sequencing

Two hundred forty-eight samples (241 samples and 7 controls) were prepared for shotgun metagenomic sequencing according to Illumina Nextera XT library preparation kit guidelines, using unique dual indexes for multiplexing with the Nextera XT index kit (Illumina). Following indexing and clean-up, samples were pooled to an equimolar concentration of 1 nM. Samples were sequenced in two pools, the first pool containing 98 samples on an Illumina NextSeq 550 sequencing platform with a V2 kit, and the second containing 150 samples on an Illumina NextSeq 2000 sequencing platform with a P3 chip, at the Teagasc DNA Sequencing Facility, using standard Illumina sequencing protocols. Four blank controls were included, one contained PBS that was used for the washing steps during sample preparation of the milk pellets, and the other three were blank extraction controls for the extraction kits that were used. Negligible amounts of DNA were obtained from these controls, which resulted in low numbers of reads, indicating that kit contamination was not an issue. These reads were assigned to very low abundances of *Pseudomonas* or *Lactococcus*, which were not removed as these two genera were present in actual samples. Three inter-run controls that had identical compositions were included in the sequencing runs to establish that no variability was introduced and no inter-run variations were identified.

### Bioinformatic analysis

Default parameters were applied for all the bioinformatic tools unless otherwise specified. Quality checks and adapter trimming were performed with FastQC (0.11.8) and cutadapt (2.6) and host reads were aligned to the bovine genome (*Bos taurus*) and removed with Bowtie2 (2.4.4). Taxonomic classification was performed with Kraken2 (2.0.7) (32) using the Genome Taxonomy Database (release 89) which contains Bacteria and Archaea (33, 34). The alphabet suffix after some genus names (e.g., *Pseudomonas_E*) indicates genera are either polyphyletic according to the database reference tree or are subdivided based on taxonomic rank normalization according to the GTDB reference tree. SUPER-FOCUS (35) was used to predict the microbiological functional potential of shotgun reads, through the alignment of reads against a reduced SEED database (36) using DIAMOND (37), with results classified into subsystems (sets of protein families with similar function). Resistome analysis was done using RGI (v.4.2.2), with the strict cut-off (38). Assembly of MAGs was done using metaSPAdes (3.13) (39), followed by binning with MetaBAT2 (2.12.1) (40) and quality assessment with checkM (1.0.18) (41). High-quality MAGs, of at least 90% completeness and less than 5% contamination, were assigned taxonomy with GTDB-tk (2.1.1) (42).

### Statistical analysis and data visualization

Statistical analysis and data visualization were performed in R (4.1.2) (43). All data were cleaned, analyzed, and visualized in R with ggplot2, tidyverse, and ggpubr packages (44, 45). Kruskal-Wallis and pairwise Wilcoxon rank sum tests with Benjamini-Hochberg *P*-value correction were used to compare sampling seasons and locations. Microbiota diversity analysis was performed with the vegan package (46), and beta diversity was calculated as Bray-Curtis metrics, visualized in a principal coordinate analysis plot. The “adonis” function from the vegan package was used to calculate the PERMANOVA to determine differences in composition of the community between groups of samples (number of permutations = 999). Redundancy analysis was also done with vegan and visualized using the ggord package (47). The “multiplatt” function from the indicspecies package was used to identify taxa that were significantly associated with particular seasons and sampling locations, by calculating Pearson’s phi coefficient of association and correcting for unequal group sizes using the parameter “r.g” (48). Pearson’s correlation was measured with the R base function, cor, and visualized using ggcorrplot (49). The core microbiota was all the taxa present in all raw milk samples analyzed, as previously defined in other literature (2, 4).

## Data Availability

Sequence data generated during the current study have been deposited in the European Nucleotide Archive under the accession number PRJEB55911. High-quality MAGs can be found at https://doi.org/10.6084/m9.figshare.25021028.v1.

## SUPPLEMENTAL MATERIAL

Supplemental material msystems.01290-23-s0001.pdf Tables S1-S4 and Figure S1.

ASM does not own the copyrights to Supplemental Material that may be linked to, or accessed through, an article. The authors have granted ASM a non-exclusive, world-wide license to publish the Supplemental Material files. Please contact the corresponding author directly for reuse.
