# Supplementary material for: Seasonal and geographical impact on the Irish raw milk microbiota correlates with chemical composition and climatic variables
Source: mSystems. 2024 Mar 6;9(4):e01290-23. doi: 10.1128/msystems.01290-23 (PMC11019797; doi:10.1128/msystems.01290-23)
Supplement: Supplemental material — Tables S1-S4 and Figure S1. [file msystems.01290-23-s0001.pdf]

SUPPLEMENTAL MATERIAL

TABLE S1. Species assignment into 5 categories (environment, host, pathogen, spoilage or technologically relevant).

Species	Average relative abundance (%)	Category	References
Acinetobacter albensis	3.90	Environment	Krizova et al. (2015), Doughari et al. (2011), Sutthiwong et al. (2023), Guo et al. (2021), Syromyatnikov et al. (2022), Wu et al. (2019), Nguyen et al. (2020), Quigley et al. (2013)
Acinetobacter bohemicus	0.16		
Acinetobacter guillouiae	0.29		
Acinetobacter johnsonii	0.42		
Acinetobacter sp002135415	0.34		
Arthrobacter_A sp002909415	0.38		
CAG-791 sp900101015	0.24		
Moraxella_A aerosaccus	0.21		
Pararhizobium sp001426685	0.39		
Rothia sp002418375	0.24		
Anaplasma phagocytophilum	0.47	Host	Silaghi et al. (2018), Taponen et al. (2019), Parks et al. (2017), Braem et al. (2013), Tan et al. (2014)
Cellulosimicrobium aquatile	0.18		
Murimonas intestini	0.14		
RUG420 sp900317985	0.22		
Kocuria atrinae	0.16		
Kocuria sp002295155	0.19		
Corynebacterium xerosis	0.49	Pathogen	Oliver et al. (2005), Rohrbach et al. (1992), McAuley et al. (2014), Zastempowska et al. (2016), Jayarao et al. (2006), Ruusunen et al. (2013), Bianchi et al. (2013), Fox et al. (2018), Van Bokhorst-van de Veen et al. (2015), Fernandes (2009)
Macrococcus caseolyticus	0.69		
Staphylococcus aureus	2.13		
Streptococcus suis_A	0.82		
Streptococcus uberis	1.47		
Trueperella pyogenes	0.76		
Brochothrix thermosphacta	0.43	Spoilage	Júnior et al. (2018), Ternström et al. (1993), Van Bokhorst-van de Veen et al. (2015), Fernandes (2009), Quigley et al. (2013), Gopal et al. (2015)
Flavobacterium frigidarium	1.46		
Microbacterium sp001425645	1.08		
Pseudomonas_E fluorescens_AH	0.48		
Pseudomonas_E fluorescens_BA	0.33		
Pseudomonas_E fragi	0.15		
Pseudomonas_E fragi_B	1.46		
Pseudomonas_E helleri	0.16		
Pseudomonas_E lactis	0.23		
Pseudomonas_E lundensis	0.71		
Pseudomonas_E lurida	2.86		
Pseudomonas_E proteolytica	0.58		
Pseudomonas_E sp002966775	0.85		
Pseudomonas_E sp900187495	1.62		
Pseudomonas_E veronii	0.78		
Psychrobacter sp002352555	0.96		
Serratia proteamaculans_B	0.60		
Bifidobacterium mongoliense	0.17		

Bifidobacterium pseudolongum_A	0.45	Technologically relevant	Quigley et al. (2013), Perin et al. (2019), Li et al. (2018)
Carnobacterium maltaromaticum	0.23		
Lactococcus lactis	2.46		
Lactococcus lactis_E	0.16		
Lactococcus piscium_C	0.14		
Lactococcus raffinolactis	0.86		
Lactococcus raffinolactis_A	0.18		
Leuconostoc lactis_A	0.42		
Leuconostoc mesenteroides	0.32		

TABLE S2. High-quality MAGs, with taxonomy assigned using GTDB-tk with the quality determined by checkM. They can be found at https://doi.org/10.6084/m9.figshare.25021028.v1.

Season	Location	GTDB-tk assignment*	Completeness	Contamination
spring	A	s__*Brochothrix thermosphacta*	99.23	0.08
spring	A	**s__*Microbacterium maritypicum***	99.72	3.15
spring	A	s__*Rothia* sp002418375	99.16	0
spring	A	g__*Acinetobacter*	90.46	1.32
spring	A	s__*Acinetobacter albensis*	99.57	0
spring	A	s__*Lactococcus_A raffinolactis*	93.43	1.59
spring	B	s__*Pararhizobium* sp001426685	92.49	1.32
spring	B	s__*Pararhizobium* sp001426685	97.73	1.23
spring	B	**s__*Pseudomonas_E bubulae***	95.21	0.89
spring	B	s__*Rothia* sp002418375	96.49	0.37
spring	B	s__*Staphylococcus aureus*	93.98	0.3
spring	B	**s__*Lactococcus petauri***	97.92	0.31
spring	C	s__*Pararhizobium* sp001426685	99.49	1.01
spring	C	s__*Pseudomonas_E proteolytica*	96.69	0
spring	C	s__*Trueperella pyogenes*	95.46	0.24
spring	D	s__*Leuconostoc lactis_A*	98.62	3.83
spring	D	s__*Pararhizobium* sp001426685	96.13	0.56
spring	D	s__*Acinetobacter albensis*	99.31	0.11
spring	D	**s__*Pseudomonas_E saxonica***	90.01	0.41
spring	D	**s__*Lactococcus_A laudensis***	93.44	0.34
spring	D	s__*Leuconostoc mesenteroides*	92.58	0.7
spring	D	s__*Lactococcus cremoris*	94.82	1.3
spring	D	**s__*Helcococcus ovis***	98.46	1.35
spring	D	s__*Trueperella pyogenes*	90.61	0.08
spring	D	s__*Streptococcus uberis*	97.88	0
spring	D	s__*Leuconostoc mesenteroides*	93.52	0.66
spring	E	s__*Pararhizobium* sp001426685	96.8	0.14
spring	E	s__*Pararhizobium* sp001426685	98.21	0.68
spring	E	s__*Lactococcus_A raffinolactis*	91.36	0.53
spring	E	s__*Pseudomonas_E helleri_A*	97.61	2.64
spring	E	s__*Acinetobacter albensis*	98.82	0.67
spring	F	s__*Trueperella pyogenes*	98.15	0
spring	F	s__*Leuconostoc mesenteroides*	99.63	0
spring	F	**s__*Lactococcus_A laudensis***	96.91	0
spring	F	s__*Leuconostoc mesenteroides*	96.23	2.01
spring	F	s__*Bifidobacterium mongoliense*	95.71	0.14
spring	F	**s__*Pseudomonas_E saxonica***	99.12	0
spring	F	s__*Leuconostoc mesenteroides*	94.53	0.11
spring	F	**s__*Lactococcus_A laudensis***	92.43	0.38
spring	G	s__*Pararhizobium* sp001426685	95.22	0.27
spring	G	s__*Leuconostoc mesenteroides*	98.74	0
spring	G	s__*Rothia* sp002418375	98.11	1.01
spring	G	**s__*Lactococcus_A laudensis***	92.61	0.31
spring	G	**s__*Lactococcus cremoris***	94.65	0.46
spring	H	s__*Rothia* sp002418375	93.83	0.54

spring	H	***s__Pseudomonas_E bubulae***	92.43	0
spring	H	***s__Lactococcus_A laudensis***	95.85	1.89
spring	I	***s__Pseudomonas_E bubulae***	97.24	0
spring	I	***s__Lactococcus cremoris***	98.87	1.13
spring	I	*s__Acinetobacter albensis*	96.6	0.69
spring	I	*s__Leuconostoc lactis_A*	90.94	1.1
summer	A	*s__Macrococcus_B caseolyticus*	95.34	0.8
summer	A	*s__Staphylococcus aureus*	96.86	1.7
summer	A	*s__Lactococcus_A raffinolactis*	99.62	1.32
summer	A	*s__Macrococcus_B caseolyticus*	99.47	0.26
summer	A	*s__Acinetobacter albensis*	98.88	1.12
summer	C	*s__Macrococcus_B caseolyticus*	91.95	0.25
summer	C	***s__Lactococcus cremoris***	90.27	1.36
summer	D	***s__Lactococcus cremoris***	100	0.13
summer	D	***g__Specibacter***	96.13	0.61
summer	E	*s__Macrococcus_B caseolyticus*	90.18	0.4
summer	F	*s__Flavobacterium frigidarium*	93.38	0
summer	F	*s__Macrococcus_B caseolyticus*	92.68	1.64
summer	F	*s__Flavobacterium frigidarium*	97.56	0.41
summer	G	*s__Leuconostoc mesenteroides*	94.3	0.55
summer	G	*s__Acinetobacter albensis*	98.89	0.61
summer	G	*s__Leuconostoc mesenteroides*	98.82	0.53
summer	G	***s__Lactococcus cremoris***	96.92	0.45
summer	G	*s__Acinetobacter albensis*	94.15	0.82
summer	G	***s__Streptococcus dysgalactiae***	94.63	0.14
summer	G	***s__Lactococcus_A carnosus***	93.31	1.36
summer	H	***s__Lactococcus cremoris***	95.01	3.43
summer	H	***s__Lactococcus_A carnosus***	94.54	0.14
summer	H	*s__Staphylococcus aureus*	90.53	1.52
summer	H	*s__Leuconostoc mesenteroides*	100	0
summer	H	*s__Carnobacterium maltaromaticum*	100	0
summer	H	***s__Pseudomonas_E paracarnis***	90.86	1.03
summer	I	*s__Macrococcus_B caseolyticus*	90.03	0.11
summer	I	*s__Lactococcus_A raffinolactis*	90.51	0.44
summer	I	***s__Moraxella_A* sp002478835**	93.38	0
summer	I	*g__Lactococcus_A*	96.02	0.32
summer	I	*s__Acinetobacter albensis*	99.62	1.7
summer	I	*s__Streptococcus uberis*	98.41	1.69
summer	I	*s__Acinetobacter albensis*	94.48	0.55
autumn	A	*s__Acinetobacter albensis*	94.48	0.55
autumn	C	*s__Leuconostoc mesenteroides*	98.69	2.85
autumn	C	***s__Lactococcus cremoris***	93.5	0.66
autumn	C	***g__Frigoribacterium***	95.6	3.08
autumn	D	***s__Lactococcus cremoris***	99.22	3.15
autumn	D	*s__Acinetobacter albensis*	97.79	0.18
autumn	E	*s__Rothia* sp002418375	99.63	1.79
autumn	F	*s__Leuconostoc mesenteroides*	99.36	0
autumn	F	***s__Epilithonimonas bovis***	98.87	0.38

autumn	F	*s__Leuconostoc mesenteroides*	96.76	0.82
autumn	F	***s__Lactococcus_A laudensis***	99.25	0.47
autumn	F	*s__Bifidobacterium mongoliense*	97.22	0.8
autumn	F	*s__Flavobacterium frigidarium*	93.12	0
autumn	F	***s__Psychrobacter immobilis_F***	99.76	3
autumn	F	***s__Pseudomonas_E bubulae***	93.07	0.67
autumn	F	*s__Acinetobacter* sp002135415	96.57	0
autumn	G	*s__Leuconostoc mesenteroides*	99.81	3.15
autumn	G	*s__Leuconostoc lactis_A*	99.25	0.75
autumn	G	*s__Acinetobacter albensis*	98.54	0
autumn	G	***s__Microbacterium maritypicum***	98.58	2.3
autumn	G	*s__Acinetobacter albensis*	97.03	0.55
autumn	H	***s__Lactococcus cremoris***	100	0
autumn	H	*g__Acinetobacter*	99.47	1.06
autumn	H	***s__Streptococcus agalactiae***	100	0.53
autumn	H	*s__Acinetobacter albensis*	94.36	0.61
autumn	I	***g__Porphyromonas_A***	100	0
autumn	I	*s__Lactococcus lactis*	97.25	0
autumn	I	*s__Lactococcus_A raffinolactis*	93.65	3.66
autumn	I	*s__Leuconostoc mesenteroides*	96.23	0.5
autumn	I	*s__Macrococcus_B caseolyticus*	98.11	0.19
autumn	I	***s__Streptococcus ruminantium***	97.55	0.98
autumn	I	***s__Microbacterium maritypicum***	99.44	0
autumn	I	*s__Acinetobacter albensis*	98.75	1.62
autumn	I	***s__Lactococcus cremoris***	91.47	0.47
autumn	I	*s__Acinetobacter albensis*	99.12	0.38
autumn	I	***s__Pseudomonas_E bubulae***	99.51	3
winter	A	*g__Acinetobacter*	96.13	1.1
winter	A	*s__Acinetobacter albensis*	98.94	0.53
winter	A	***s__Lactococcus cremoris***	97.44	0.53
winter	A	*s__Acinetobacter albensis*	95.02	0.27
winter	B	***s__Pseudomonas_E bubulae***	97.54	0.08
winter	C	***s__Pseudomonas_E bubulae***	97.36	1.19
winter	D	*s__Acinetobacter albensis*	92.42	0
winter	D	*s__Rothia* sp002418375	97.74	1.07
winter	F	*s__Acinetobacter albensis*	93.39	0.61
winter	F	***s__Lactococcus_A laudensis***	97.81	4.92
winter	F	*s__Lactococcus_A raffinolactis*	95.08	2.51
winter	F	*s__Acinetobacter albensis*	92.26	2.42
winter	F	***s__Leuconostoc rapi***	95.58	1.66
winter	F	*s__Leuconostoc mesenteroides*	99.5	0.38
winter	F	***s__Lactococcus_A laudensis***	93.07	0.58
winter	F	***s__Lactococcus_A laudensis***	95.53	0
winter	F	*s__Flavobacterium frigidarium*	97.34	0
winter	F	*s__Leuconostoc mesenteroides*	95.31	1.82
winter	G	*s__Acinetobacter albensis*	94.89	0.38
winter	G	*s__Lactococcus_A raffinolactis*	99.62	1.19
winter	G	*s__Leuconostoc lactis_A*	98.91	0.53

winter	G	*s__Lactococcus_A raffinolactis*	94.25	0.57
winter	G	*s__Acinetobacter albensis*	98.26	0.27
winter	H	***s__Pseudomonas_E bubulae***	98.74	1.13
winter	H	*s__Streptococcus uberis*	94.03	0
winter	I	*s__Pseudomonas_E fragi*	91.02	1.95
winter	I	*s__Staphylococcus aureus*	96.13	1.66
winter	I	***s__Pseudomonas_E bubulae***	95.58	0.55
winter	I	*s__Acinetobacter albensis*	95.64	1.26
winter	I	*s__Brochothrix thermosphacta*	98.31	0.36
winter	I	*s__Acinetobacter albensis*	95.28	0.5
winter	I	*s__Rothia* sp002418375	95.8	0
winter	I	*s__Lactococcus_A raffinolactis*	93.61	0.27

*Taxa in bold text indicates high-quality MAGs recovered that were not detected in the classification of short reads using Kraken2.

TABLE S3. Relative abundances of genera and subsystem level 1 functions that differed significantly by sampling season.

Genera	Season[#]			
	spring	summer	autumn	winter
Pseudomonas_E	22.77	5.41*	12.02*	31.90
Lactococcus	6.48	10.41^	6.03	6.91
Rothia	1.70	4.63^	2.57	0.51*
Pararhizobium	5.93^	0.01*	0.00*	0.00*
Staphylococcus	0.90	1.59^	1.18	1.13
Macrococcus	0.40	3.21^	0.63	0.04
Streptococcus	0.87	1.46^	1.06	1.01
Microbacterium	0.95	1.16	1.02	0.30*
Psychrobacter	1.22*	0.30*	0.53	0.55
Bifidobacterium	0.96	0.34*	0.41*	0.75
Flavobacterium	0.68	1.01^	0.17	0.49
Moraxella_A	0.23	1.44^	0.49	0.04*
Carnobacterium	0.30	0.75^	0.21	0.77^
Brochothrix	0.35	0.22	0.05*	1.16^
Anaplasma	0.25	0.86^	0.26	0.05*
CAG 791	0.39	0.11*	0.39	0.46

Subsystem level 1 functions	Season			
	spring	summer	autumn	winter
Arabinose Sensor and transport module	0.000966^	0.000779	0.000516	0.000696
Carbohydrates	13.424	14.229^	13.361	12.409*
Cell Wall and Capsule	4.9328	4.984	4.926	5.126^
Clustering-based subsystems	5.737^	5.627	5.596	5.616
Cofactors, Vitamins, Prosthetic Groups, Pigments	6.424	6.542	6.636^	6.574
Dormancy and Sporulation	0.141	0.139	0.179^	0.146
Fatty Acids, Lipids, and Isoprenoids	3.172	3.189	3.328	3.392^
Nitrogen Metabolism	1.269	1.103*	1.196	1.224
Phages, Prophages, Transposable elements	0.062	0.086^	0.0628	0.0656
Potassium metabolism	1.064	0.974*	1.045	1.169^
Respiration	3.418	3.376	3.574^	3.371*
Transcriptional regulation	0.153	0.156	0.153	0.139*
Virulence	4.643	4.208*	4.438	4.506
Virulence, Disease and Defence	0.506*	0.658^	0.549	0.548

[#] Mean abundance values

* denotes that sampling location was significantly lower

^ denotes that location had a significantly higher mean relative abundance compared to other locations

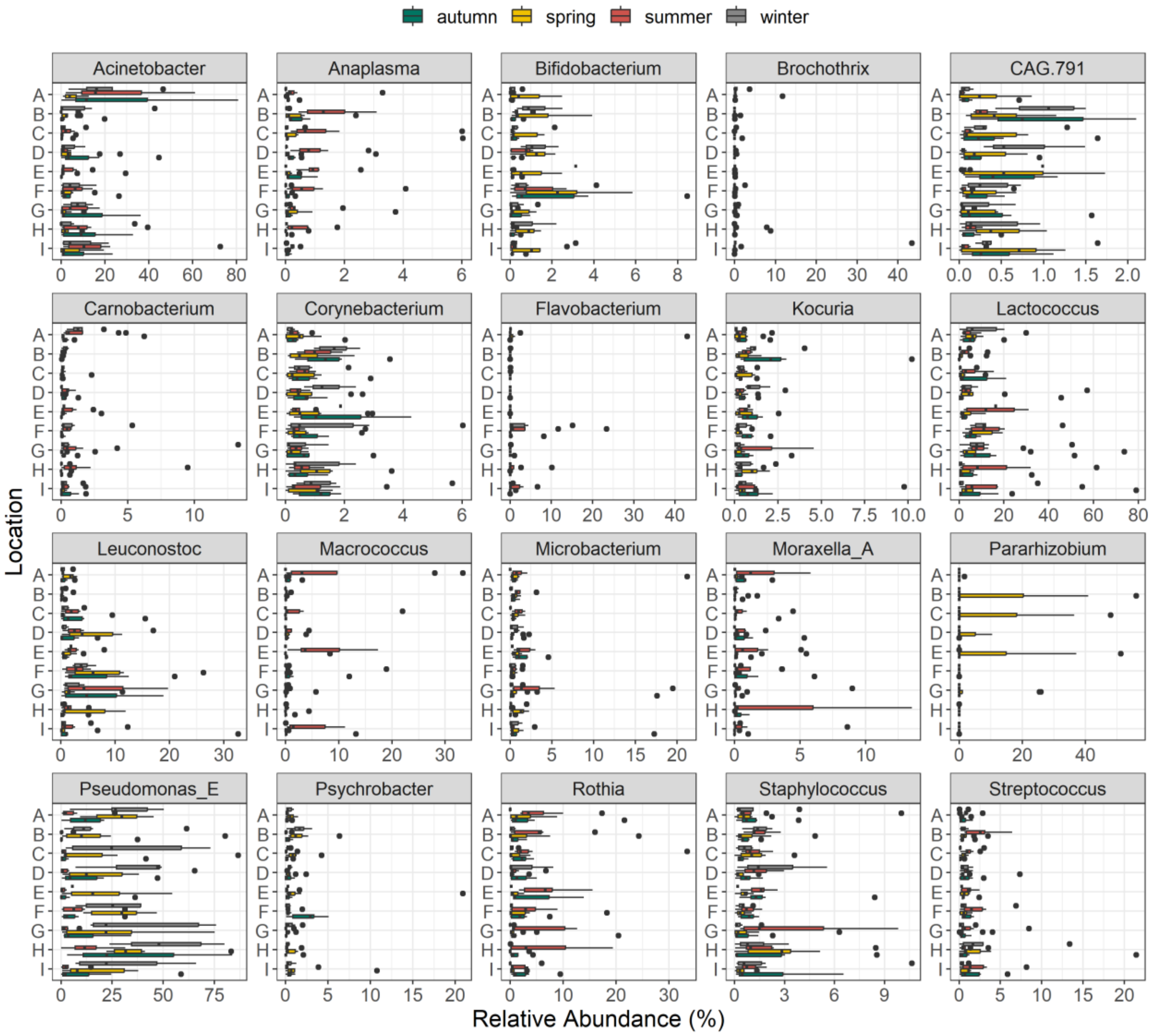

FIGURE S1. Relative abundances of the top 20 genera in boxplots by location and season.

TABLE S4. Relative abundances of genera and subsystem level 1 functions that differed significantly by sampling location.

Genera	Sampling location[#]								
	A	B	C	D	E	F	G	H	I
Pseudomonas_E	17.43	10.85	14.29	16.24	10.29	16.09	20.05	32.59^	15.02
Lactococcus	6.71	1.85*	4.20*	7.06	6.71	10.19	12.77	7.35	10.97
Acinetobacter	19.34^	3.95	1.87*	6.18	2.93	5.41	6.66	8.00	10.06
Leuconostoc	0.76	0.28*	2.13	3.42	1.48	5.22^	4.35^	1.66	2.76
Pararhizobium	0.07	4.54	3.13	0.96	4.38	0.00	1.75	0.00*	0.00
Macrococcus	2.92	0.11*	1.05	0.55	2.30	1.41	0.29	0.32*	1.76
Microbacterium	1.29	0.59	0.56	0.60	1.17	0.33*	1.92	0.40*	1.20
Corynebacterium	0.35	1.19^	0.65	0.70	1.03	0.88	0.46	0.81	1.11^
Kocuria	0.44	1.27^	0.45	0.57	0.68	0.44	0.61	0.56	0.97
Bifidobacterium	0.26*	0.73	0.36	0.74	0.53	1.66^	0.29*	0.40	0.53
Flavobacterium	1.77	0.03	0.02	0.02	0.02	2.49^	0.06	0.49	0.56
Moraxella_A	0.84^	0.16	0.37	0.47	0.78	0.64	0.42	1.22	0.47
Carnobacterium	1.08^	0.10	0.17	0.25	0.38	0.42	0.92	0.64	0.40
Brochothrix	0.68^	0.05	0.10	0.05	0.02	0.12	0.15	0.64	1.83^
Anaplasma	0.21	0.58	0.70^	0.49	0.45	0.33	0.32	0.20	0.10
CAG 791	0.14*	0.66^	0.31	0.28	0.46	0.22	0.22	0.27	0.39

Subsystem level 1 functions	Sampling location								
	A	B	C	D	E	F	G	H	I
Carbohydrates	12.01*	13.91	13.79	13.72	14.15	13.59	13.76	12.04*	13.75
Central metabolism	0.18	0.22	0.22	0.19	0.24^	0.20	0.21	0.18*	0.20
Clustering-based subsystems	5.67	5.72	5.60	5.55	5.82^	5.54	5.60	5.74	5.62
Cofactors, Vitamins, Prosthetic Groups, Pigments	6.57	6.66^	6.63	6.43	6.49	6.64	6.42	6.46	6.55
Dormancy and Sporulation	0.13	0.21^	0.15	0.14	0.16	0.13	0.14	0.15	0.15
Fatty Acids, Lipids, and Isoprenoids	3.61^	3.22	3.21	3.19	3.10	3.14	3.19	3.39	3.30
Metabolism of Aromatic Compounds	1.36	1.29	1.16	1.18	1.13	1.09	1.14	1.39^	1.16
Miscellaneous	2.96	3.07	3.20	3.15	3.13	3.22^	3.15	2.96	3.07
Nitrogen Metabolism	1.33	1.27	1.23	1.19	1.17	1.11*	1.09*	1.25	1.15
Nucleosides and Nucleotides	3.39	3.82^	3.58	3.60	3.78	3.61	3.56	3.26*	3.68
Phages, Prophages, Transposable elements	0.06	0.05*	0.06	0.07	0.07	0.08	0.09	0.08	0.08
Phages, Prophages, Transposable elements, Plasmids	0.93	0.79*	0.87	0.92	1.04	1.00	1.03	0.97	1.00
Photosynthesis	0.02	0.04^	0.03	0.02	0.04	0.02	0.02	0.02*	0.02
Protein Metabolism	8.00	8.63^	8.78	8.39	8.42	8.40	8.19	7.55*	8.42
Secondary Metabolism	0.04	0.06^	0.06	0.05	0.05	0.04	0.04	0.05	0.04*
Sulphur Metabolism	1.63^	1.38	1.28	1.36	1.28	1.35	1.36	1.59^	1.41
Transcriptional regulation	0.14*	0.16	0.15	0.15	0.16	0.15	0.15	0.15	0.15
Virulence	4.80^	4.13	4.23	4.65	4.21	4.50	4.50	4.65	4.41
Virulence, Disease and Defence	0.54	0.45*	0.51	0.57	0.54	0.58	0.63	0.67	0.61

[#] Mean abundance values have been rounded to 2 decimal places.
***** denotes that sampling location was significantly lower
^ denotes that location had a significantly higher mean relative abundance compared to other locations

References

Bianchi DM, Barbaro A, Gallina S, Vitale N, Chiavacci L, Caramelli M, Decastelli L. 2013. Monitoring of foodborne pathogenic bacteria in vending machine raw milk in Piedmont, Italy. Food Control 32:435-439.

Braem G, De Vliegher S, Verbist B, Piessens V, Van Coillie E, De Vuyst L, Leroy F. 2013. Unraveling the microbiota of teat apices of clinically healthy lactating dairy cows, with special emphasis on coagulase-negative staphylococci. Journal of Dairy Science 96:1499-1510.

Doughari HJ, Ndakidemi PA, Human IS, Benade S. 2011. The ecology, biology and pathogenesis of Acinetobacter spp.: an overview. Microbes and environments 26:101-112.

Fernandes R. 2009. Microbiology handbook: dairy products. Royal Society of Chemistry.

Fox EM, Jiang Y, Gobius KS. 2018. Key pathogenic bacteria associated with dairy foods: On-farm ecology and products associated with foodborne pathogen transmission. International Dairy Journal 84:28-35.

Gopal N, Hill C, Ross PR, Beresford TP, Fenelon MA, Cotter PD. 2015. The prevalence and control of Bacillus and related spore-forming bacteria in the dairy industry. Frontiers in microbiology 6:1418.

Guo X, Yu Z, Zhao F, Sun Z, Kwok L-Y, Li S. 2021. Both sampling seasonality and geographic origin contribute significantly to variations in raw milk microbiota, but sampling seasonality is the more determining factor. Journal of Dairy Science 104:10609-10627.

Jayarao BM, Donaldson SC, Straley BA, Sawant AA, Hegde NV, Brown J. 2006. A survey of foodborne pathogens in bulk tank milk and raw milk consumption among farm families in Pennsylvania. Journal of dairy science 89:2451-2458.

Júnior JR, De Oliveira A, Silva FdG, Tamanini R, De Oliveira A, Beloti V. 2018. The main spoilage-related psychrotrophic bacteria in refrigerated raw milk. Journal of Dairy Science 101:75-83.

Krizova L, Maixnerova M, Sedo O, Nemec A. 2015. Acinetobacter albensis sp. nov., isolated from natural soil and water ecosystems. International journal of systematic and evolutionary microbiology 65:3905-3912.

Li N, Wang Y, You C, Ren J, Chen W, Zheng H, Liu Z. 2018. Variation in raw milk microbiota throughout 12 months and the impact of weather conditions. Scientific reports 8:2371.

Mcauley CM, McMillan K, Moore SC, Fegan N, Fox EM. 2014. Prevalence and characterization of foodborne pathogens from Australian dairy farm environments. Journal of dairy science 97:7402-7412.

Nguyen TT, Wu H, Nishino N. 2020. An investigation of seasonal variations in the microbiota of milk, feces, bedding, and airborne dust. Asian-Australasian Journal of Animal Sciences 33:1858.

Oliver SP, Jayarao B, Almeida R. Foodborne pathogens, mastitis, milk quality, and dairy food safety, p 3-27. In (ed),

Parks DH, Rinke C, Chuvochina M, Chaumeil P-A, Woodcroft BJ, Evans PN, Hugenholtz P, Tyson GW. 2017. Recovery of nearly 8,000 metagenome-assembled genomes substantially expands the tree of life. Nature microbiology 2:1533-1542.

Perin LM, Pereira JG, Bersot LS, Nero LA. 2019. The microbiology of raw milk, p 45-64, Raw milk. Elsevier.

Quigley L, O'Sullivan O, Stanton C, Beresford TP, Ross RP, Fitzgerald GF, Cotter PD. 2013. The complex microbiota of raw milk. FEMS microbiology reviews 37:664-698.

Rohrbach BW, Draughon FA, Davidson PM, Oliver SP. 1992. Prevalence of Listeria monocytogenes, Campylobacter jejuni, Yersinia enterocolitica, and Salmonella in bulk tank milk: risk factors and risk of human exposure. Journal of Food Protection 55:93-97.

Ruusunen M, Salonen M, Pulkkinen H, Huuskonen M, Hellström S, Revez J, Hänninen M-L, Fredriksson-Ahomaa M, Lindström M. 2013. Pathogenic bacteria in Finnish bulk tank milk. Foodborne pathogens and disease 10:99-106.

Silaghi C, Nieder M, Sauter-Louis C, Knubben-Schweizer G, Pfister K, Pfeffer M. 2018. Epidemiology, genetic variants and clinical course of natural infections with Anaplasma phagocytophilum in a dairy cattle herd. Parasites & vectors 11:1-13.

Sutthiwong N, Lekavat S, Dufossé L. 2023. Involvement of Versatile Bacteria Belonging to the Genus Arthrobacter in Milk and Dairy Products. Foods 12:1270.

Syromyatnikov MY, Nesterova EY, Gladkikh MI, Tolkacheva AA, Bondareva OV, Syrov VM, Pryakhina NA, Popov VN. 2022. High-Throughput Sequencing as a Tool for the Quality Control of Microbial Bioformulations for Agriculture. Processes 10:2243.

Tan H, Deng Q, Cao L. 2014. Ruminant feces harbor diverse uncultured symbiotic actinobacteria. World Journal of Microbiology and Biotechnology 30:1093-1100.

Taponen S, McGuinness D, Hiitiö H, Simojoki H, Zadoks R, Pyörälä S. 2019. Bovine milk microbiome: a more complex issue than expected. Veterinary Research 50:1-15.

Ternström A, Lindberg AM, Molin G. 1993. Classification of the spoilage flora of raw and pasteurized bovine milk, with special reference to Pseudomonas and Bacillus. Journal of Applied Bacteriology 75:25-34.

van Bokhorst-van de Veen H, Minor M, Zwietering M, Groot MN. 2015. Microbial hazards in the dairy chain: A literature study.

Wu H, Nguyen QD, Tran TT, Tang MT, Tsuruta T, Nishino N. 2019. Rumen fluid, feces, milk, water, feed, airborne dust, and bedding microbiota in dairy farms managed by automatic milking systems. Animal Science Journal 90:445-452.

Zastempowska E, Grajewski J, Twaruzek M. 2016. Food-borne pathogens and contaminants in raw milk-a review. Annals of Animal Science 16:623.
